# Flow orientation as a critical parameter in nanoscale membrane filtration for optimising small extracellular vesicle isolation

**DOI:** 10.1039/d6na00035e

**Published:** 2026-03-27

**Authors:** Sadeka Nujhat, Hannah S. Leese, Madeleine A. Strickland, Mirella Di Lorenzo, Sandhya Moise

**Affiliations:** a Department of Chemical Engineering, University of Bath Bath BA2 7AY UK s.moise@bath.ac.uk h.s.leese@bath.ac.uk; b Centre for Bioengineering and Biomedical Technologies (CBio), University of Bath Bath BA2 7AY UK

## Abstract

Membrane filtration of biological nanoparticles typically employs tangential or perpendicular flow geometries, each presenting inherent performance trade-offs between membrane flux and fouling. While these configurations involve established limitations in transmembrane pressure, fouling, and particle damage, how intermediate inlet angles quantitatively modulate these effects at the microscale remains unexplored. Here, we combine computational fluid dynamics with experimental validation to systematically investigate how inlet microchannel orientation relative to a nanoporous alumina membrane (0°, 30°, 60°, and 90°) governs the separation performance of small extracellular vesicles (sEVs) – sub-200 nm particles that serve as promising liquid biopsy targets for ovarian cancer detection. Simulations show that intermediate flow angles (30° and 60°) produce filtration fluxes between those of perpendicular and tangential configurations, reflecting modified near-membrane hydrodynamics, while potentially improving filtrate composition. Using OVCAR3 ovarian cancer cell-derived sEVs, we demonstrate that a 60° inlet angle generates a distinct hydrodynamic regime favourable for sEV membrane-based filtration. Devices with inlet orientation at 60° (i) suppress contaminants of >100 nm by 3-fold compared to perpendicular flow, (ii) retain comparable sEV recovery compared to tangential flow (*p* < 0.05), and (iii) enhance biomarker detectability, with CD9 increasing up to 6-fold and CA125 up to 2.5-fold relative to conventional geometries. Together, these results identify flow orientation as a key design parameter in nanoscale membrane bioseparation, showing that intermediate geometries create distinct transport behaviours. They also provide experimentally validated foundations for advancing nanofiltration-enabled sEV isolation, directly relevant to improving ovarian cancer diagnostics given the value of sEV-associated CA125 as an early-stage biomarker.

## Abstract 2

A 60° inlet channel angle to the membrane reduces contaminant co-filtration while maximising sEV recovery and biomarker signals compared to conventional flow directions, providing a design strategy for microfluidic nanofiltration of sEVs.

## Graphical abstract

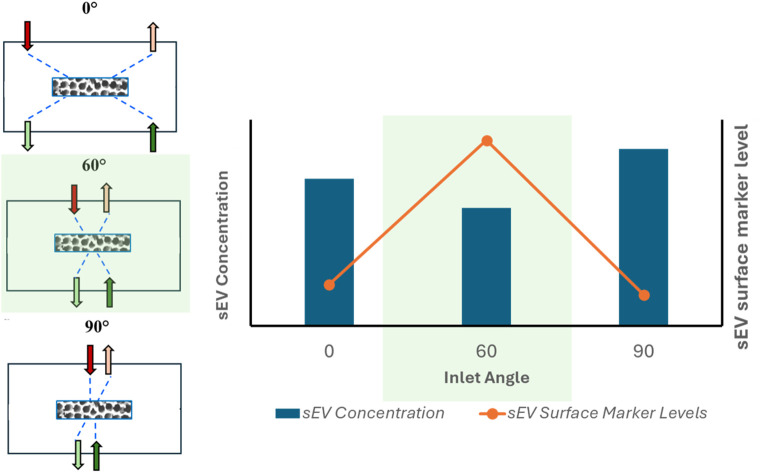

## Introduction

Ovarian cancer is the deadliest gynaecological cancer and fifth leading cause of cancer-related deaths in women. This outcome is due to over 70% of cases being diagnosed in late stages, where the 5 year survival rate can be as low as 20%; in contrast, early-stage diagnosis offers over a 90% survival rate.^1,2^ The absence of symptoms in early stages and then occurrence of only nontypical symptoms coupled with the lack of reliable screening tests delay ovarian cancer diagnosis. The CA125 serum level is the current gold standard for ovarian cancer detection, but its clinical utility is limited by poor sensitivity and specificity.^3–6^ In contrast, CA125 carried on small extracellular vesicles (sEVs), as a tumour-derived, vesicle-associated form of the biomarker, shows promise for improved diagnostic performance.^7–9^ sEVs are a diverse and highly heterogeneous population of phospholipid bilayer-bound particles, typically less than 200 nm in diameter.^10–12^ Their ability to encapsulate and protect biomolecular cargo from enzymatic degradation makes sEVs a robust source of disease-specific biomarkers, reflecting the physiological condition of their cell of origin.^9,13,14^ However, isolating sEVs routinely from complex biofluids remains technically challenging due to their small size, fragility, low abundance, and coexistence with other vesicle subtypes and contaminants, such as lipoproteins and protein aggregates.^12,15,16^

Ultracentrifugation, the most conventional technique for sEV separation, is laborious and time-consuming and often co-isolates impurities.^17–19^ Size exclusion chromatography (SEC) offers higher purity but suffers from low yield and poor scalability.^20,21^ Precipitation methods are simple but introduce chemical contaminants that can interfere with downstream analyses^22,23^ while immunoaffinity capture improves specificity but is costly and limited by antibody performance and scalability.^24–26^ Membrane-based filtration has emerged as a promising alternative to traditional isolation strategies, offering operational simplicity and scalability.^27–29^ Recent studies have demonstrated the potential in both discovery and validation of cancer biomarkers using nanoscale microfluidic platforms, such as ExoTIC,^30,31^ Exosearch^32^ and Exodisc.^33^ Yet, fouling, shear-induced vesicle damage, and inconsistent filtrate quality remain significant bottlenecks for membrane-based filtration systems.^30,31^ Tangential flow filtration (TFF), which directs flow parallel to the membrane surface (0° angle), can mitigate fouling by generating shear forces that limit particle deposition^34,35^ while retaining a stable permeate flux over prolonged operation with low transmembrane pressures.^34,36^ However, because the feed stream flows tangentially rather than directly towards the membrane, the driving force for particle transport across the membrane is reduced. This, in turn, reduces filtration efficiency for nanoscale particles such as sEVs, especially when their concentrations are low or heterogeneous.^35^ In contrast, perpendicular filtration, where flow is at a 90° angle to the membrane, subjects particles to maximal hydrodynamic drag toward the membrane, which enables high particle flux across the membrane. However, this configuration is prone to rapid fouling and cake formation,^37–39^ and higher transmembrane pressures to retain permeate flux can lead to vesicle deformation and rupture.^40,41^ While intermediate angles could theoretically balance tangential and perpendicular flows by accessing intermediate hydrodynamic regimes that balance shear, permeation, residence time and pressure variables, the behaviour of nanoscale sEVs under varying flow conditions is uncertain.^42^ Traditionally, studies on sEV isolation *via* membrane nanofiltration have focussed on optimising the flow rate, transmembrane pressure and membrane characteristics rather than inlet angle geometry because it is assumed that TFF is generally an optimal system for sEV nanofiltration.^35,43,44^ However, since tangential and dead-end flow each have their advantages and limitations, here we hypothesise that an intermediate angle might achieve an intermediate hydrodynamic regime that balances the extremes of dead-end and tangential flow and can substantially influence vesicle recovery, size selectivity, and biomarker integrity, providing quantitative guidance for the design of microfluidic nanofiltration systems for diagnostic applications.

Material selection is crucial for membrane performance: conventional polymeric membranes such as polyacrylonitrile (PAN) and polyvinyl chloride (PVC) often lack chemical stability and fouling resistance.^39,45^ In contrast, ceramic membranes, such as nanoporous alumina, are chemically inert and mechanically robust and feature highly uniform, tuneable pore structures, supporting reusability, customisability and reliable performance.^40,46,47^ Combining nanoporous alumina membrane filtration with microfluidics, the latter being well-suited for point-of-care (POC) settings,^48–51^ presents a compelling strategy for achieving efficient, scalable, and user-friendly sEV enrichment, particularly for clinical applications.^48–51^

Here, we have established a new platform for sEV nanofiltration that explores intermediate inlet microchannel orientations to nanoporous membranes, aiming to balance sEV recovery, size selectivity, biomarker retention, and membrane fouling. We implement this approach using microfluidic devices with nanoporous alumina membranes to isolate sEVs from OVCAR3 cells, a well-established high-grade serous ovarian carcinoma model.^52,53^ First, computational fluid dynamics simulations in COMSOL are used to evaluate how inlet angle influences solute transport across the membrane. These candidate angles are then experimentally tested to determine their impact on sEV concentration, size distribution, and retention of ovarian cancer-associated (CA125) and general sEV (CD9) biomarkers.

## Results and discussion

### Identifying the optimal inlet channel orientation for membrane nanofiltration

**Fig. 1:**
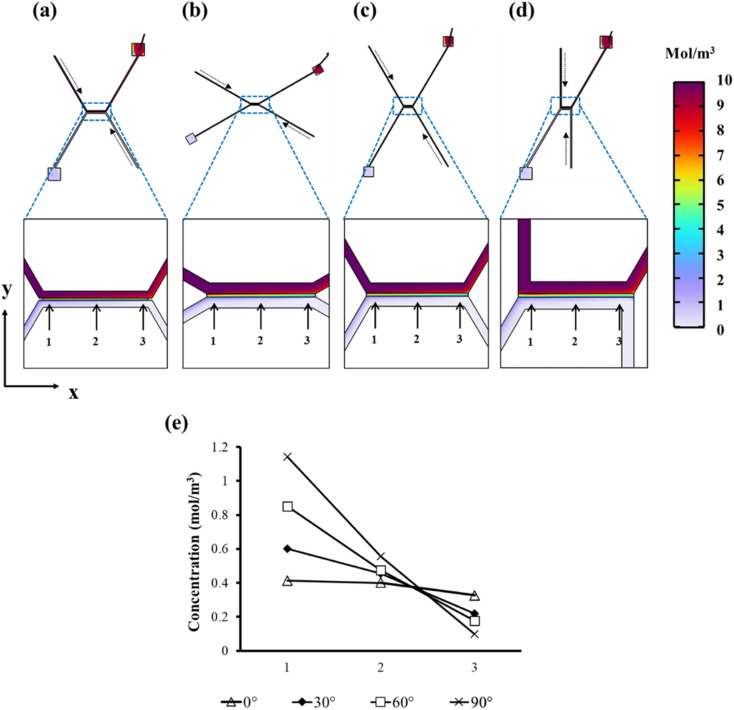
Computational analysis of the effect of angle on filtration: COMSOL computational fluid dynamic simulations of the sample concentration following nanofiltration at 0° (a), 30° (b), 60° (c) and 90° (d) at 30 s post-initiation of flow using alumina (membrane) and blood (sample) as materials, with creeping flow fluid characteristics and mass transfer in porous media (membrane). Dashed arrows indicate the direction of flow; solute concentration analysis was performed downstream of the membrane at each angle, at 30 seconds from initiating flow at equidistant points (arrows at 1, 2 and 3 points) along the *x*-axis (direction of flow of the sample) (e).

We computationally assessed the optimal inlet sample flow rate to achieve maximal transmembrane flux of solutes. Computational simulations show that an inlet flow rate of 5 µL min^−1^ achieves a higher membrane flux of the solutes, noted by the higher sample concentration downstream of the membrane (Fig. 1), than that at 1 or 10 µL min^−1^ for all angles studied (Fig. S1). However, most of the solute in the injected sample is collected in the retentate and only ∼10% permeates through the membrane (Fig. 1a–d). As expected, the solute concentration, downstream of the membrane, is highest on the sample feed side and decreases as the sample moves along the direction of flow (left to right) (Fig. 1e). The microchannel perpendicular (90°) to the membrane had the highest concentration at point 1 (closest to the sample feed inlet) and also the highest total overall transport (area under the curve: 1.20 mol m^−3^ at 90°) followed by a 17.5% drop at 60° (0.99 mol m^−3^), 27.5% at 30° (0.87 mol m^−3^), and then 35.8% at the tangential configuration at 0° (0.77 mol m^−3^) (Fig. 1e). While the perpendicular concentration appears to have the highest filtration efficiency, in terms of total solute transport, the current model does not account for other factors, such as pore size and membrane fouling. For instance, the 90° angle has a higher fluid flow to the membrane, resulting in forcefully pushing the sample across the membrane, potentially damaging the bioparticles (*e.g.* sEV bursting, deforming or being debrided of surface markers) whilst fouling the membrane and limiting filtration efficiency.^54,55^ The 0° angle also has a lateral fluid flow reducing sEV movement to the membrane, reducing permeate flux and therefore limiting sample filtration efficacy, albeit with reduced membrane fouling.^56^ The 30° and 60° angles may present as middle points to achieve more efficient filtrations by adjusting the fluid flow direction for a reduced risk of membrane fouling and improved sEV integrity.

### Fabrication of nanoporous membrane-integrated microfluidic devices

This work is the first demonstration of using the ESCARGOT technique^57^ to create angled microfluidic channels in PDMS structures. By modifying the original ESCRAGOT technique which uses acetone wash to dissolve the ABS filament, the filament was removed using tweezers, significantly reducing fabrication time and minimising solvent usage, whilst providing the option to also substitute ABS with a more sustainable filament material in the future. Furthermore, as the filament shape remained intact after removal, the filaments were reused as a mold for additional microchannel fabrication, further conserving materials and time.

**Fig. 2:**
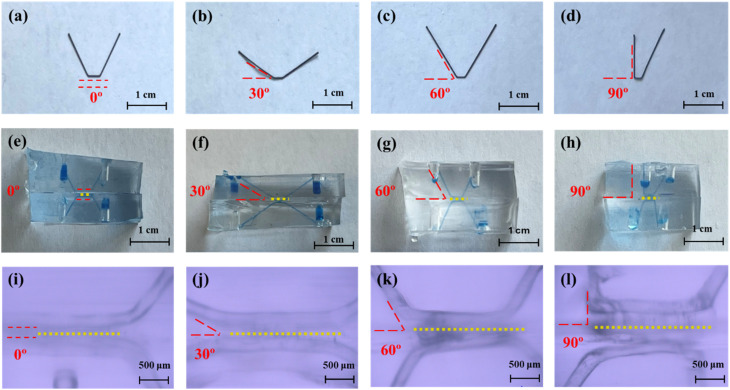
Device fabrication: 3D printed ABS filaments at 0° (a), 30° (b), 60° (c) and 90° (d) angles. (e–h) Microfluidic devices at each angle with a blue dye for microchannel visualisation and yellow dashed lines representing membrane location. Microscope images of microchannels at 0° (i), 30° (j), 60° (k) and 90° (l) angles with yellow dashed lines representing membrane location.

The 3D printed ABS filaments were accurate in dimensions with a consistent thickness of 200 µm and angle sizes of 0°, 30°, 60° and 90° (Fig. 2a–d). The anodised alumina membranes were successfully integrated within the angled devices (yellow dashed lines in Fig. 2e–h), and the microscope images show retention of the geometry and thickness after manual extraction of the ABS filaments (Fig. 2i–l).

### Inlet microchannel orientation to the membrane defines the filtrate sEV concentration

**Fig. 3:**
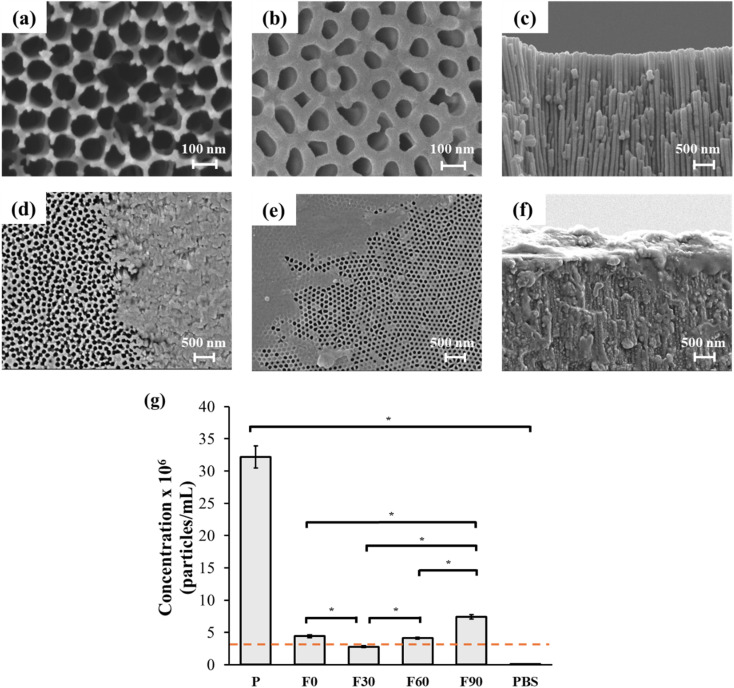
Membrane nanofiltration: SEM images of membranes pre (a–c) and post (d–f) filtration, anodised (a and d), etched (b and e) and cross-section (c and f) sides. Concentration variations following filtration of conditioned media (denoted by ‘P’) obtained from 48-hour OVCAR3 culture. The sample was filtered across a 100 nm pore alumina membrane at 0° (F0), 30° (F30), 60° (F60) and 90° (F90) inlet angles at 5 µL min^−1^ with comparison to computational simulation prediction (dashed orange line) (g). Data are presented as *n* = 3 ± standard error of the mean; *p < 0.05; statistical analysis was performed using ANOVA repeated measures.

Anodised membrane pores were measured to be 100.7 ± 6.7 nm on the anodised side (Fig. 3a and d) and 97.9 ± 13.2 nm on the etched side of the membrane (Fig. 3b and e). Devices fabricated with integrated 100 nm alumina membranes (Fig. 3a–c) with their monolithic pores (Fig. 3c) were assessed for their filtration efficiency using sEVs derived from unfiltered OVCAR3 cell-conditioned media (parent sample). There is clear evidence of membrane fouling post-filtration (Fig. 3d–f). The anodised side of the membrane (Fig. 3a and d) is on the parent sample inlet side, and the etched side (Fig. 3b and e) faces the PBS flow side. Post filtration, membranes in each device qualitatively show a similar extent of fouling on both sides of the membrane, regardless of the inlet flow angle, as shown in the representative SEM images in Fig. 3. The post filtration anodised side of the membrane (Fig. 3d) shows a thick and textured fouling on the surface, indicating a mixture of small and larger vesicles from the parent sEV sample. In contrast, the etched side of the post filtration membrane (Fig. 3e) shows a smoother and thinner layer of fouling (most pores are visible through the fouling layer), implying the flow of a ‘clearer’ sample, such as PBS. Additionally, as shown by the cross-section SEM image of the post filtration membrane (Fig. 3f), fouling also occurred within the pore channels of the membrane in addition to surface fouling.

The parent sEV sample (P) had the highest particle concentration while PBS (control) had the lowest concentration, as expected (Fig. 3g). Filtrates, F0, F30, F60, and F90 (where the numbers refer to the microchannel angle), parent (P) and PBS samples had significantly different (*p* < 0.05) particle concentrations among each other except for F0 and F60 (Fig. 3g). The experimental percentage filtration (between 9% and 23%) aligns reasonably with the computational prediction (Fig. 1a–d) of ∼10% (Fig. 3g, dashed orange line). The simulation showed a trend of decreasing percentage filtration with decreasing angle size (Fig. 1e), which was also reflected experimentally (Fig. 3g) with F90 having the most concentrated filtrate (23%) and a gradual percentage filtration decrease to F60 (13%) and F30 (9%). F0 was expected to have the lowest percentage filtrate according to the computation simulation; however, the increased residence time in this device to reach proper tangential flow and minimal fouling shifted vesicle filtration to a higher percentage filtration (14%).^35^

One limitation of the computational model is that it did not account for sample loss including membrane fouling, which, as shown in experiments (Fig. 3a–f), occurs in practice. The inlet microchannel angle at 60° presented the highest loss of particle concentration when compared to its retentate (Fig. S2) followed by the 0° setup. This difference can be attributed to more fluid flow directed to the membrane in the 60° device, which promotes higher deposition of sEVs on the membrane, and longer residence time in the 0° device, both of which contribute to loss of the sample.^58^ Conversely, although the 90° configuration would typically be expected to show the most fouling, it instead produced a higher vesicle concentration value of filtrate and retentate combined, compared to the parent sample (Fig. S2). This result may be due to the high concentration polarization, causing aggregates to fragment into smaller particles, artificially inflating particle counts, while rapid cake layer formation at the membrane likely created a physical barrier that prevented further filtration and retained particles in the retentate flow.^59^ Overall, the 90° angle yielded the highest total particle concentration in the filtrate, consistent with our computational simulations. The 90° angle's filtration efficiency of 23% aligns with other reported work on sEVs from cell-conditioned media undergoing dead-end nanofiltration using 70 and 110 nm pore size alumina membranes.^40^

### Microchannel orientation configuration provides superior ovarian cancer sEV detection

**Fig. 4:**
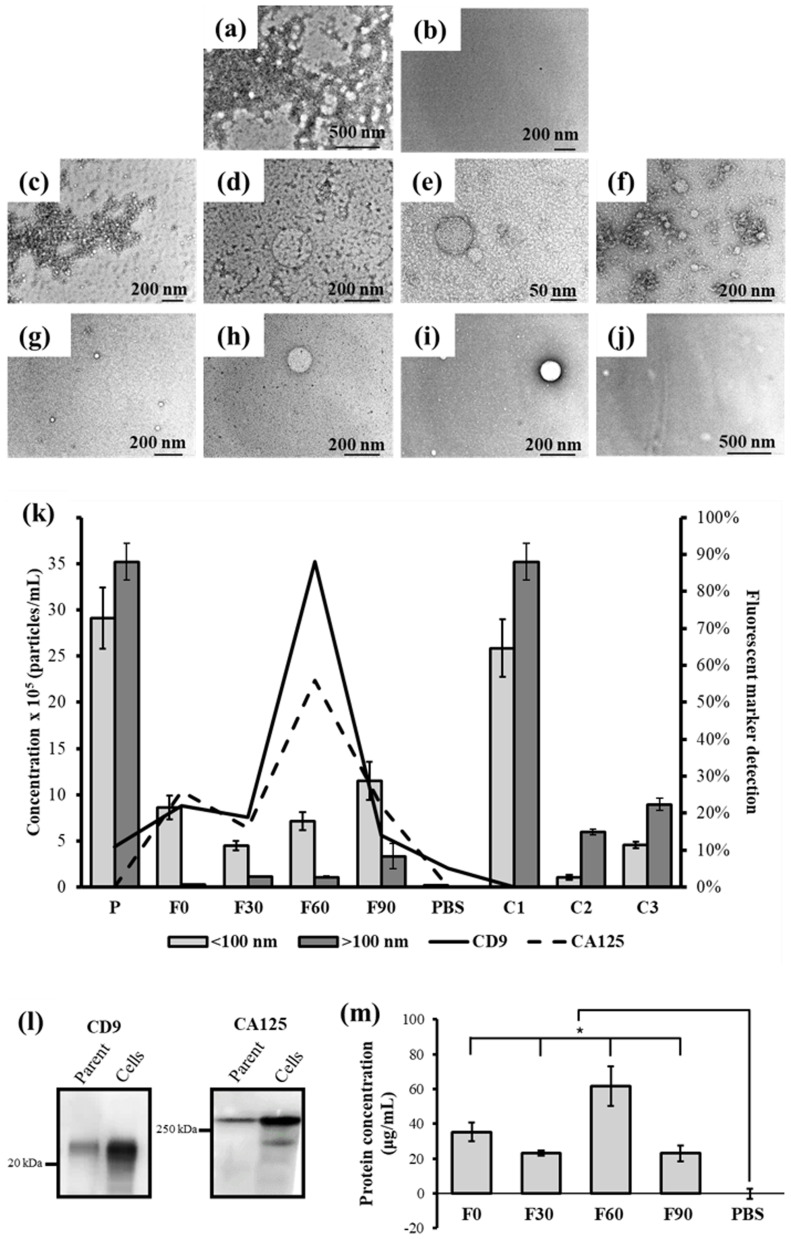
TEM images of unfiltered OVCAR3 conditioned media following 48 h culture of cells (denoted by ‘P’) (a), PBS (b), retentates of 0° (c), 30° (d), 60° (e), and 90° (f) and filtrates of 0° (g), 30° (h), 60° (i) and 90° (j) of P, filtered using angled inlet channels (0°, 30°, 60°, and 90°) within microfluidic devices at 5 µL min^−1^; (k) concentration of vesicle subpopulations as measured through nanotracking analysis (<100 nm and > 100 nm), normalised intensity of vesicles expressing CD9 and CA125 comparison across the OVCAR3 conditioned media (P), filtrates (F0, F30, F60 or F90), retentates (R0, R30, R60 or R90), PBS, unstained OVCAR3 conditioned media control (C1), incubated antibody solution (C2), and IgG isotope control (C3). (l) Western blot for CA125 and CD9 detection in OVCAR3 cells and OVCAR3 conditioned media; (m) BCA analysis of protein detection in filtrates compared to PBS. Normalised protein concentration was analysed using the calibration curve in Fig. S5 (low). *n* = 3, error bars refer to standard error of the mean; *p < 0.05. Statistical analysis was performed using Welch's ANOVA with the Games Howell *post hoc* test.

TEM images confirm that the parent sample (Fig. 4a) contains the highest concentration of extracellular vesicles, consistent with the NanoSight particle analysis (Fig. 3g). As expected, PBS shows no detectable particles (Fig. 4b). All retentates, R0, R30, R60, and R90 (Fig. 4c–f), display lower vesicle concentrations than the parent sample, likely due to vesicles passing through the membrane into the filtrate and vesicle deposition on the microchannel walls and membrane surface (fouling). Most vesicles in the retentates are larger than 100 nm, indicating effective size exclusion by the membrane cut off pore size. An exception is seen in R90, where vesicles smaller than 100 nm are still visible, likely due to increased fouling impeding proper filtration. The filtrates, F0, F30, F60, and F90 (Fig. 4g–j), predominantly contain vesicles smaller than 100 nm, especially in F0. F30 and F60 contain few vesicles slightly larger than the membrane pore size, which may be explained by the flexible lipid bilayer allowing vesicles to deform and pass through narrower pores where sufficient pressure is present.^60^ In F90, vesicles appear less defined, suggesting that high filtration pressures may have compromised vesicle integrity, although most remain under 100 nm in diameter.^41^

To quantify sEV size distribution after filtration, vesicle concentrations above and below 100 nm in each filtrate were compared (Fig. 4k). The 90° filtration yielded the highest proportion of particles under 100 nm, followed by the 0°, 60°, and 30° angles. However, the 90° setup also produced the greatest number of vesicles above 100 nm (22.3%), whereas the 0° configuration had the fewest (2.9%). Filtrates from the 30° and 60° microchannel orientations showed similar but minimal concentrations of vesicles larger than 100 nm (20.0% and 13.2%, respectively), showing ∼3-fold higher size specificity to the 0° configuration. Overall, based on filtrate composition, the 0° angle appears to provide the most precise size-selective filtration which aligns with the expected behaviour of nanoporous membranes, where minimal perpendicular flow reduces the likelihood of larger vesicles being forced through the pores. Heinemann and Vykoukal (2017)^41^ suggest an sEV filtration protocol combining dead-end (to remove large particles) followed by tangential flow filtration (to remove non-EV associated biomolecules and debris) to achieve size-defined sEV populations. Although applied sequentially, this strategy conceptually aligns with our finding that an angled filtration, which combines the perpendicular and tangential flow components, in a single filtration step, improves sEV size-specific recovery and purity. Notably, very few studies explicitly assess size precision in sEV filtration as most prioritise total recovery,^61^ and none compare various inlet microchannel orientations to the membrane other than TFF and dead-end flow, highlighting the significance of our work.

Western blot analysis confirms the presence of EVs and expression of ovarian cancer biomarker CA125 in both OVCAR3 cells and the parent sample (Fig. 4l). When comparing the concentrations of OVCAR3-secreted extracellular vesicles expressing CD9 and CA125 in the filtrates (Fig. 4m), F60 has the highest intensity percentage of vesicles expressing both surface markers, 88% expression of CD9 and 56% of CA125, which are 6.3-fold and 3.5-fold higher than those of the parent sample, respectively. Of note, CD9 and CA125 are not necessarily related as not all CA125^+^ sEVs contain CD9 and *vice versa*.^62^ The rest of the filtrates showed significantly lower intensity of both marker expression with F0 and F90 expressing higher CA125 levels (29% and 22%, respectively) than CD9 (22% and 14%, respectively). This result is also supported by the BCA findings where F60 shows the highest protein concentration amongst all filtrates (Fig. 4l). The control samples (Fig. 4m: C1 being the parent sample without any secondary antibody incubation and C2 being primary and secondary antibody solution, without any sEV sample) show 0% CA125 and CD9 detections as expected. PBS shows 0% CA125 detection and 5% CD9 detection which is negligible as it could have arisen from cross-reactions between antibodies or non-specific binding.^63^

F90 has the highest concentration of sub-100 nm particles in the sample, suggesting that most particles may not be sEVs, but fragments of larger vesicles broken due to the increased pressure of the angle filtration through the membrane (also observed as irregular structures seen in Fig. 4j). On the other hand, F0, also containing sub-100 nm particles in the sample with slightly higher CD9 expression levels to F90, may be explained by the fact that CD9 is also found in larger EVs.^64^ Overall, the fluorescence nanotracking analysis of CD9 (Fig. S3) revealed particularly higher concentrations in the filtrates than in the retentates for the 30° and 60° configurations. The 0° angle showed only a slight increase in the filtrate compared to the retentate, while the 90° angle exhibited a lower CD9 concentration in the filtrate than in the retentate, further proving the inefficiency of dead-end filtration due to fouling. CA125 appears to be more detectable with every subsequent filtration of the parent sample (Fig. 4m), due to removal of contaminants resulting in a ‘cleaner’ exposure of filtrate sEVs than the parent samples as seen from the TEM images (Fig. 4a–j). This indicates that the improved fluorescent signal may be associated with a higher degree of sample purity. In fact, fluorescence nanotracking analysis shows an evidently higher CA125 concentration detection in all filtrates compared to their retentate (Fig. S4).

Another contributing factor may be sample ‘clarity’: as shown in Fig. 4a, the parent sample contains a busy background of various particles and debris-like structures (*e.g.* protein and lipid aggregates, larger vesicles, and cell by-products) that can obscure antibody fluorescence signals and reduce apparent marker intensity^65^ – an established issue, which is why filtered buffers are urged in immunostaining protocols to minimise background interference.^66,67^ The 0° and 30° angles may not provide sufficient sample contact with the membrane to reduce contaminant co-filtration. This hypothesis is supported by the presence of ‘dark spots’ visible in the F0 and F30 filtrates (Fig. 4g–h), which are absent in F60 (Fig. 4i). Moreover, the retentates R0 and R30 (Fig. 4c–d) show retention of impurities that are not seen in R60 (Fig. 4e), suggesting that these contaminants settle onto the membrane when the microchannel inlet is at 60° to the nanoporous membrane, producing a ‘cleaner’ retentate and filtrate for fluorescence detection, which is also supported by the 60° configuration exhibiting the highest calculated fouling (36%, Fig. S2). Similar aggregates observed in R90 may be attributed to rapid cake layer formation, which limits filtration and traps these impurities within the retentate stream. This was proven by the fluorescence nanotracking analysis of CD9 (Fig. S3d), which showed a higher marker concentration in the retentate than in the filtrate. The reduced marker intensity observed in F90 may result from extensive sEV accumulation on the membrane surface or vesicle rupture under high transmembrane pressures, leading to marker loss.^38,68,69^ Supporting this, TEM imaging of F90 (Fig. 4j) revealed fewer defined vesicle-like structures, in contrast to the clear vesicular morphology observed in filtrates from the other angles. Hence, 60° angle filtration offers the most ideal filtrate for ovarian cancer marker detection *via* sEVs. This suggests that the 60° angle strikes an effective balance between TFF and dead-end filtration, optimising vesicle recovery while minimising shear-induced damage and membrane fouling. In contrast, the comparatively low CD9 and CA125 levels at 0° and 30° angles may reflect significant co-filtration of contaminants that impede efficient biomarker detection,^65^ an issue observed not only in marker detection but also in any fluorescence detection analysis, emphasising sample preparation and matrix-matched calibration to improve accuracy.^70^ However, for the 90° extensive membrane fouling in combination with vesicle rupture under high transmembrane pressures could have led to sEVs and, hence, biomarker loss.^61^ Although many report TFF superiority to dead-end sEV isolation methods, most lack sEV-specific biomarker detection comparisons between the different filtration configurations.^71^ Huang *et al.*, (2019)^72^ demonstrated that CA125 can be well detected in sEVs derived from ovarian cancer patient plasma albeit at markedly lower levels than in plasma. This discrepancy may stem from the use of commercial isolation kits, which often yield low-purity preparations and can compromise fluorescence-based marker detection in sEVs, further emphasising the need for sEV isolation optimisation for clinical outcomes.

Although adjusting flow orientation may appear conceptually straightforward, our findings highlight that its impact on nanoscale membrane transport is far from trivial. In nanofiltration microfluidic systems, vesicle behaviour is governed by a complex interplay of shear forces, pressure-driven fluid flow, near-membrane hydrodynamics and interactions, and vesicle deformability. As a result, intermediate flow orientations do not simply produce an “average” of tangential and dead-end filtration conditions; instead, they generate a distinct force landscape that uniquely modulates vesicle–membrane interactions. This helps explain why the 60° configuration produces markedly higher intact sEV recovery, improved size precision, and better biomarker detectability. Previous studies have focused almost exclusively on perpendicular and parallel flow geometries because these represent classical filtration limits that are straightforward to model and implement. By systematically probing the previously unexplored intermediate spectrum, our work reveals a previously unrecognised design parameter that influences vesicle transport and separation outcomes. These results underscore that nanoscale filtration performance cannot be reliably inferred from macroscale intuition and that microfluidic systems offer opportunities to uncover non-obvious geometries that substantially improve the biochemical quality of isolated extracellular vesicles.^73^

While our results reveal clear trends in how inlet microchannel orientation to the membrane influences sEV recovery, size-selectivity, and biomarker detection, they also highlight unanswered questions about the underlying mechanisms and broader applicability of these effects. This proof-of-concept study was limited to OVCAR3-derived sEVs, and it is essential to validate these observed angle-dependent trends for other cell types and clinically relevant biofluids such as plasma or urine. Validating these findings in patient-derived samples will be essential to realising the diagnostic potential of sEV-based CA125 detection relative to conventional serum testing. In addition, the study employed a single 100 nm nanoporous alumina membrane and a fixed microchannel geometry fabricated in PDMS. The membrane material, pore size, channel dimensions, and surface properties are all known to influence flow profiles, membrane flux, fouling behaviour, and particle–membrane interactions. Systematic evaluation of these parameters was beyond the scope of this study but represents an important direction for future work to determine how channel properties and membrane characteristics interact with inlet orientation to influence separation performance. Mechanistic insights could be strengthened by quantifying pressure drop and shear forces, enabling a clear link between inlet microchannel orientation to the membrane and vesicle recovery. In parallel, refining the computational model to particle tracing could improve predictive accuracy and guide optimal system design. Furthermore, as our observations suggest that intermediate angles minimise co-filtration of contaminants, future work should investigate sample purity and potential sEV cargo loss through proteomic or RNA profiling studies.

## Conclusion

Tangential and perpendicular inlet flow angles in microfluidic membrane nanofiltration suffer from inherent limitations in terms of membrane flux and fouling. We hypothesised that an intermediate inlet angle might achieve a unique hydrodynamic regime that balances the trade-offs between perpendicular and tangential flow which could improve sEV nanofiltration. We have developed a microfluidic nanofiltration system that enhances both recovery and biomarker detection of ovarian cancer-derived sEVs. Using a combined computational and experimental approach, we demonstrate that inlet microchannel orientation relative to the membrane critically governs nanofiltration efficiency. Specifically, an intermediate angle of 60° maximises recovery of sEVs, among the tested configurations, including CA125-positive vesicles, while minimising co-filtration of contaminants, yielding cleaner filtrates and improved downstream biomarker detection. This previously underexplored design parameter enables precise control over sEV size selectivity, biomarker preservation, and concentration, offering a practical framework for optimising microfluidic sEV isolation. These findings suggest potential value for enhancing nanofiltration-based sEV isolation strategies, which could advance sEV-based biomarker isolation, such as sEV-associated CA125, for the early detection of ovarian cancer.

## Experimental section

### Design and computational fluid dynamics (CFD) simulation

**Fig. 5:**
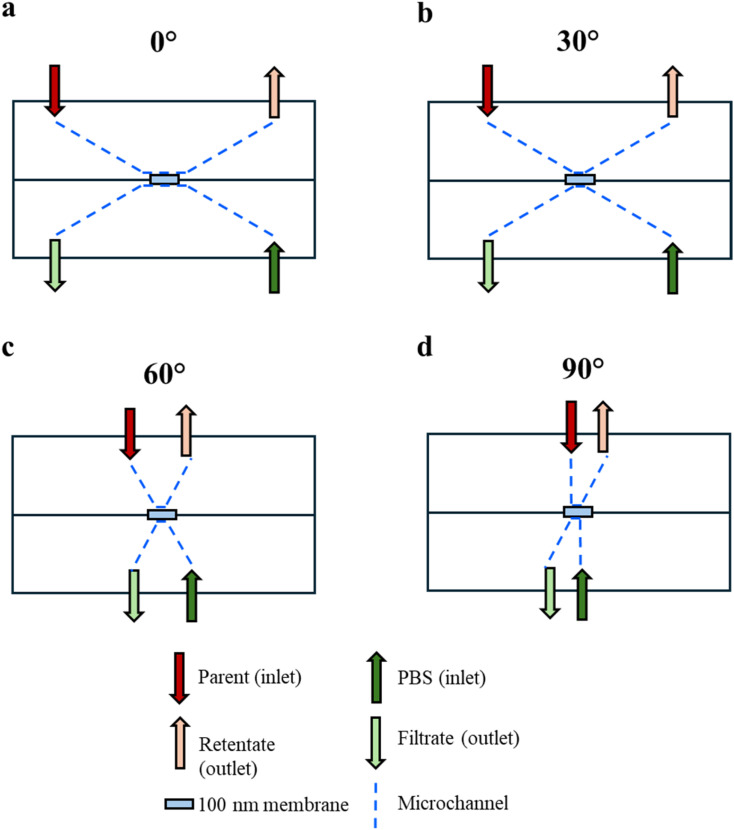
2D schematics of the filtration device systems for 0° (a), 30° (b), 60° (c) and 90° (d) angles.

The microfluidic filtration system for 0°, 30°, 60°, and 90° angles was designed as shown in Fig. 5. The sample concentration distribution across the membrane was computed using COMSOL software (COMSOL Multiphysics® 6.3, USA). The sample concentration was studied across all four angles (0°, 30°, 60° and 90°) at three different flow rates (1, 5 and 10 µL min^−1^). The microchannel geometry was initially modelled using Autodesk Fusion 360® and then imported into COMSOL where inlets, outlets, walls and membrane units were defined. Blood plasma was used as the sample material with creeping flow properties and transport of diluted species across the membrane (porous alumina material). Once results were computed, concentration values across the membrane at three equidistant points were analysed across all angles and flow rates to (a) identify the most efficient flow rate for all angles and (b) investigate concentration changes based on the inlet microchannel orientation to the membrane (Fig. 1).

### Microfluidic device fabrication

The filaments at 0°, 30°, 60° and 90° angles were designed using Autodesk Fusion 360® with 200 µm thickness and 3D printed (BambuLab P1S, China) using an Acrylonitrile Butadiene Styrene (ABS) filament (1.8 mm; Makerbot, USA) and a 0.2 mm nozzle (BambuLab P1S, China). The printing parameters were set at 0.10 mm PreciseAF, 0.1 mm layer height, minimum 0.22 mm line width, without support, and 5 mm s^−1^ initial layer speed. The 0° filament was designed with an extended residence time for filtration (3 mm *vs.* 2 mm for the rest of the angles) to ensure that the flow velocity becomes parallel (tangential or 0°) to the membrane flow as the sample is introduced upstream into the device just before the membrane surface (Fig. 5a).

The 3D printed ABS filaments were used as moulds for the microchannels in polydimethylsiloxane (PDMS; Michigan, USA) following the ‘ESCARGOT’ (Embedded SCAffold RemovinG Open Technology) technique detailed by Saggiomo and Venders (2015)^57^ where PDMS is cured around an ABS structure which is then removed to leave an empty structure of the ABS design embedded in the PDMS block. The ABS filaments in this case were used to create angled microchannels in the PDMS block as part of the microfluidic device. The devices at each angle were no larger than 30 × 20 × 20 mm (*L* × *H* × *W*) with an inlet/outlet on each side created using a 2 mm diameter biopsy punch.

Nanoporous alumina membranes were fabricated *via* anodisation of alumina wafers: using a 14 mm core punch tool and hammer, aluminium wafers were excised from a flat sheet and annealed at 500 °C for 60 minutes using an oven under a 1 kg weight. Annealed wafers were degreased by sonication in acetone for 10 min and then electropolished in a 4 : 1 solution of absolute ethanol and 37% perchloric acid held at −70 °C using a dry ice acetone bath at 20 V for 20 min. A two-step anodisation was then carried out on electropolished wafers with only a single face of the wafer exposed to oxalic acid (0.3 M) at 70 V at 5 °C for 20 min at first and then 4 h, with a phosphoric acid (0.1 M) etching step at 45 °C in between for 10 min. Following this, anodised wafers were transferred to another holder with both faces exposed where residual aluminium was removed by immersion in a solution of CuCl_2_ (0.2 M) and HCl (20%), washing frequently with deionised water until the wafer became transparent. The wafers were then finally etched again in phosphoric acid (0.1 M) to open the closed end of alumina pores.

The fabricated alumina membranes were sandwiched between two blocks of PDMS with the same angled microchannels. The anodised side of the membrane was facing the parent sample inlet, and the etched side was towards the phosphate buffered saline (PBS) flow. The membrane was sealed by curing a thin layer of PDMS using a microscope glass slide in between the blocks and the membrane and leak-proof tested using ethanol before sample filtration.

### Cell culture and parent sample collection

OVCAR3 cells (ATCC, USA; HTB-161, passage number <15) were cultured in RPMI-1640 (Gibco, USA), supplemented with 20% Foetal Bovine Serum (FBS; Gibco, USA), 1% penicillin-streptomycin (Gibco, USA) and 10 mg mL^−1^ insulin (MP Biomedicals, USA) while grown at 37 °C in a 5% CO_2_ incubator. General passage consisted of full media change twice a week and passage when cultures reached 80% confluency. Cells were counted using trypan blue (Gibco, USA) and a cell counter (DeNovix; USA). Subcultures were seeded at 20 000 cells per cm^2^.

To collect the parent sEV sample, once OVCAR3 cultures reached 80% confluency, cell culture media were altered to 20% sEV-depleted FBS (collected by ultracentrifugation of FBS for 18 h at 100 000×*g* at 4 °C). After 48 h, the conditioned media were collected and centrifuged at 150×*g* for 5 min to remove detached cells and large debris and then kept at 4 °C for a maximum of a week for filtration experiments.

### Filtration experiments

All experimental runs were performed in biological triplicate on separate devices. Identical volumes of unfiltered OVCAR3 conditioned media samples (referred to as ‘parent’) were loaded on separate devices. Identical volumes of parent sample and PBS were injected into the microfluidic device at three flow rates 1, 5 and 10 µL min^−1^ using a syringe pump (KD Scientific, USA) through the appropriate inlets (Fig. 5). Filtrate and retentate samples were collected from *n* = 3 filtrations for further analysis from the respective ports of the device.

### Scatter and fluorescence nanoparticle tracking analysis (NTA)

All samples were analysed for size distribution using a NanoSight NS300 (Malvern, UK). The speed of the syringe pump was varied between 30 and 50 AU (Arbitrary Units) and detection threshold was set between 9 and 12 to achieve 10–100 particles per frame, <5 blue crosses (not valid particle tracks) per frame and at least 7 s of exposure of each particle in the frame. 3 × 30 second captures were recorded of each sample for analysis.

For fluorescence nanotracking analysis, each sample was incubated with the same volume of 1 : 250 dilution of either CD9 antibody (ThermoFisher, USA; 10626D, lot 00998135) or CA125 antibody (AbCam, UK; ab693, lot GR3368082-3) in 1% BSA in PBS overnight under agitation at 4 °C. Samples were then washed by SEC (ImmunoStep; Spain) and fractions 5–8 were collected for fluorescent labelling. 3 µg mL^−1^ of Alexa Fluor 488 solution (ThermoFisher, USA) was used to incubate samples at a 1 : 1 volume ratio on ice for 1 h in the dark. Finally, samples were identified using the NanoSight NS300 in the light scatter mode and then with the filter wheel at 500 nm, where 3 × 30 second captures were recorded for sample analysis. Three controls were used: (i) unstained OVCAR3 conditioned media (C1), (ii) incubated antibody solution (C2), and (iii) an IgG isotope control (C3; Purified Mouse IgG1, κ Isotype Ctrl Antibody, Biologend, USA). Average concentrations (*n* = 3) for both scatter and fluorescent samples are in particles per mL ± SEM format and are calculated from the analysed data using the NTA software (version 3.4).

### Western blot

Samples were analysed for total protein concentration using a bicinchoninic acid (BCA) protein assay kit (Pierce, USA). Thirty µg of each sample were loaded in 4–20% acrylamide gels and separated at 100 V for 60 min. Protein bands were transferred to a nitrocellulose membrane at 200 mV for 90 minutes and incubated with 1 : 1000 CD9 and CA125 primary antibodies overnight under agitation at 4 °C. After secondary antibody incubation at 1 : 2000 for an hour in the dark, bands were imaged (ChemiDoc™ MP, Bio-Rad, USA) with 5 minutes of exposure.

### BCA

Twenty-five µL of sample was mixed with 200 µL BCA working reagent (Pierce, USA) and after 30 min of incubation at 37 °C, sample absorbance at 562 nm was measured. Protein concentrations were estimated using equations derived from the calibration curve (see Fig. S5).

### Transmission electron microscopy (TEM)

Ten µL of samples were placed on hydrophilic copper Formvar/carbon TEM grids for 60 s and blotted dry with filter paper, following a 10 µL deionised water wash for 1 second and blotting to dry with filter paper again. The samples were then negatively stained using 5 µL of 2% uranyl acetate for 60 s, blot dried and observed under the TEM JEM-2100Plus (JEOL, Japan).

### Field emission scanning electron microscopy (Fe-SEM)

Both sides of the nanoporous alumina membranes, anodised and pore-opened (etched), were imaged for pore size analysis and fouling (pre and post filtration). For post filtration membrane analysis, devices were cut apart to retrieve the used membrane using a scalpel to isolate two pieces of membranes (one for each side to image) that were exposed to the filtration area. Samples were degassed overnight and covered with 10 nm of chromium before being imaged using a JSM-7900 (JEOL, Japan).

### Statistical analysis and measurements

All statistical analyses were performed using IBM SPSS Statistics (version 20) with repeated measures ANOVA or Welch's ANOVA with Games Howell post-hoc tests (in case of unequal variance). All data are presented as average ± standard error of the mean (SEM). Normalised percentage fluorescent particle concentration was calculated by comparing the area under the curve, according to eqn (1).1
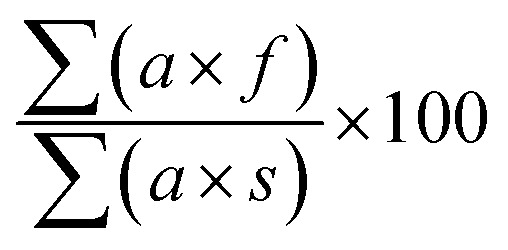
where *a* is the particle size (bin centre, nm) in nm; *f* is the fluorescent particle concentration (particles per mL); *s* is the scatter particle concentration (particles per mL). Mean anodised alumina membrane pore sizes were measured using ImageJ (version 1.54g).

## Conflicts of interest

The authors declare no conflict of interest.

## Supplementary Material

NA-OLF-D6NA00035E-s001

## Data Availability

Data for this article, including Nanosight data, are available at the University of Bath Research Data Archive at https://doi.org/10.15125/BATH-01584. Supplementary information (SI): experimental details, including nanotracking analysis graphs and table, COMSOL analysis and photographs, and BCA calibration curves. See DOI: https://doi.org/10.1039/d6na00035e.
