# Supplementary material for: Flow orientation as a critical parameter in nanoscale membrane filtration for optimising small extracellular vesicle isolation
Source: Nanoscale Adv. 2026 Mar 27;8(10):3175–87. doi: 10.1039/d6na00035e (PMC13093753; doi:10.1039/d6na00035e)
Supplement: NA-008-D6NA00035E-s001 [file NA-008-D6NA00035E-s001.pdf]

Supplementary Information (SI) for Nanoscale Advances.
This journal is © The Royal Society of Chemistry 2026

Supplementary

Flow Orientation as a Critical Parameter in Nanoscale Membrane Filtration for Optimising Small Extracellular Vesicle Isolation

*Sadeka Nujhat, Hannah S Leese, Madeleine A Strickland, Mirella Di Lorenzo, Sandhya Moise**

S1

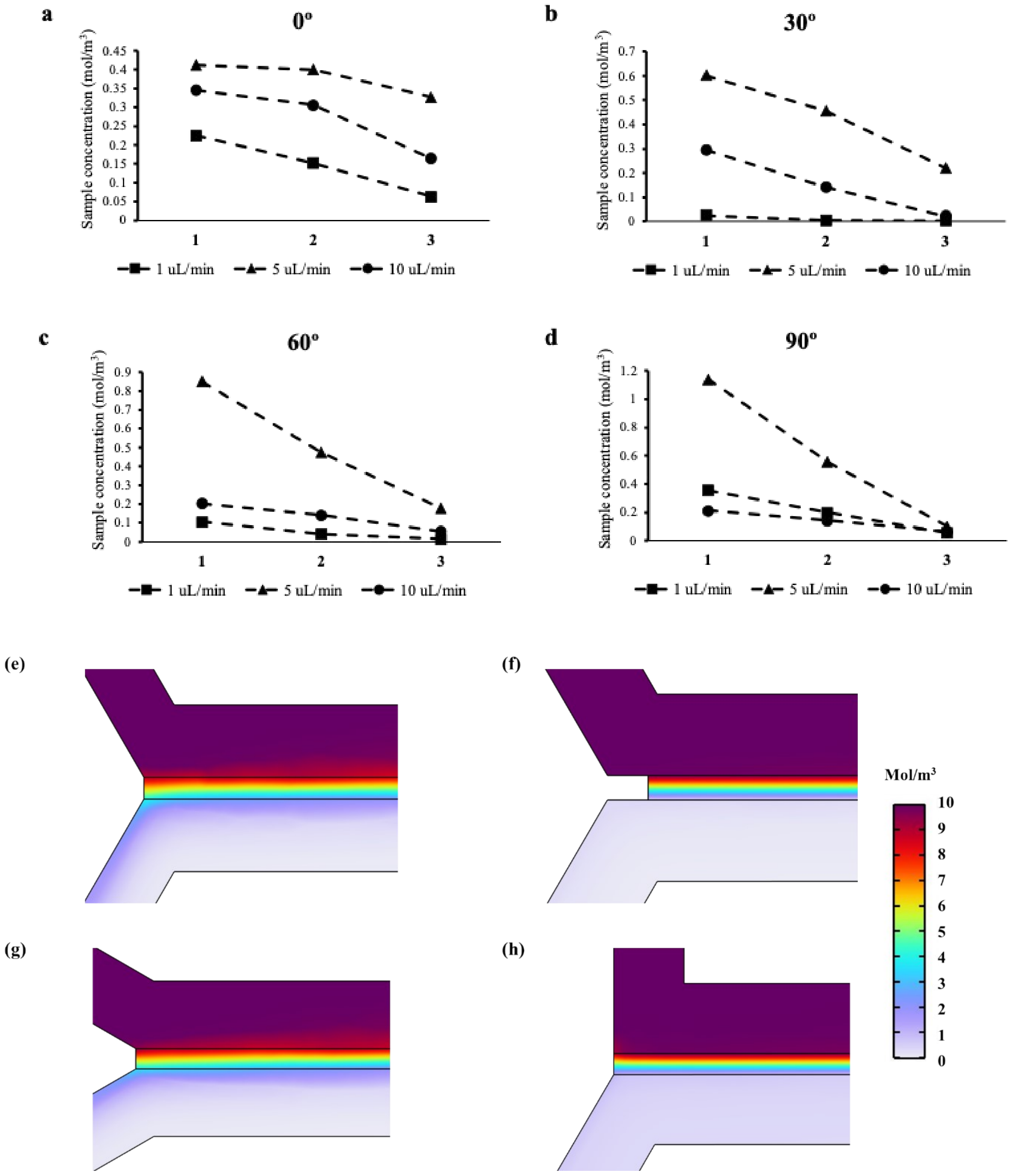

Figure S1: *Computational simulation on COMSOL for sample concentration gradient from three equidistant spots across the membrane at three different sample flow rates, 1, 5 and 10 μL min-1 at 0º (a), 30º (b), 60º (c) and 90º (d) angles for inlet microchannel orientation to the nanoporous membrane in the microfluidic device. Zoomed in panels of figure 1 showing simulation results of concentration profiles in the 0º (e), 30º (f), 60º (g) and 90º (h) angle set-ups.*

S2

a

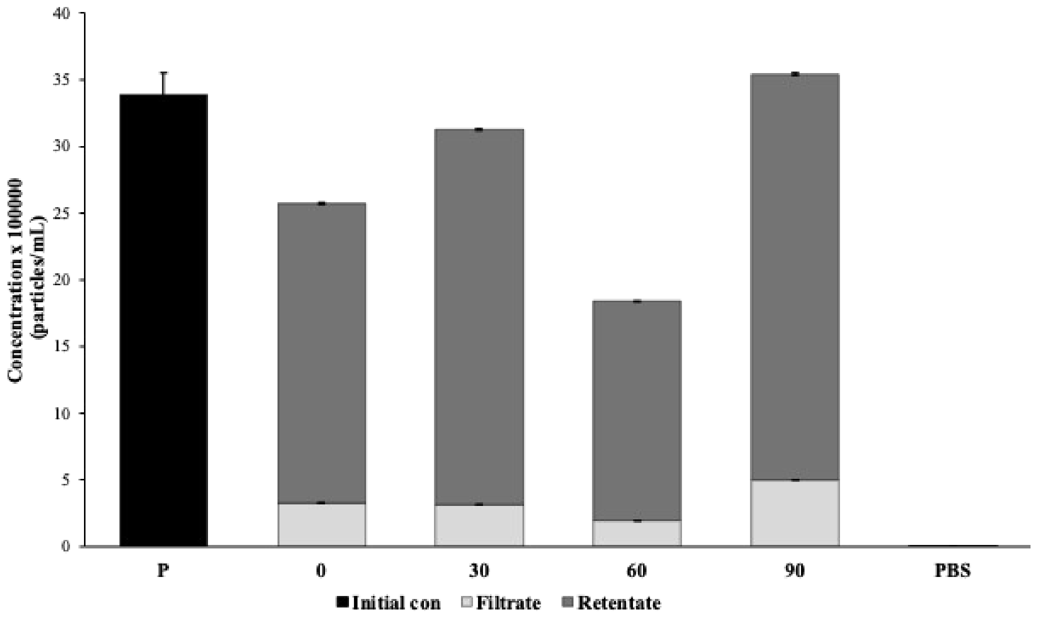

b

Sample	Initial concentration (x10³ particles/mL)	Filtrate (x10³ particles/mL)	Retentate (x10³ particles/mL)	Filtrate (%)	Retentate (%)	Loss (%)
**P**	3,215.7 ± 10.3					
**0**		443,5 ± 1.0	2,246.4 ± 137.2	14	70	16
**30**		278,4 ± 0.7	2,812.5 ± 133.5	9	87	4
**60**		410,2 ± 0.8	1,643.7 ± 101.3	13	51	36
**90**		741,5 ± 1.5	3,045.6 ± 177.5	23	95	-
**PBS**	9.6 ± 0.04					

Figure S2: *Filtrate and retentate sEV concentrations from filtrations at each inlet microchannel angle to the nanoporous membrane compared to the parent (OVCAR3 conditioned media) and PBS (buffer.) Data are presented as n = 3 ± SEM (a); Table analysing percentage loss of sEVs from comparing filtrates and retentates from filtrations at each inlet microchannel angle to the nanoporous membrane compared to the parent and PBS. Data are presented as n = 3 ± SEM (b)*

S3

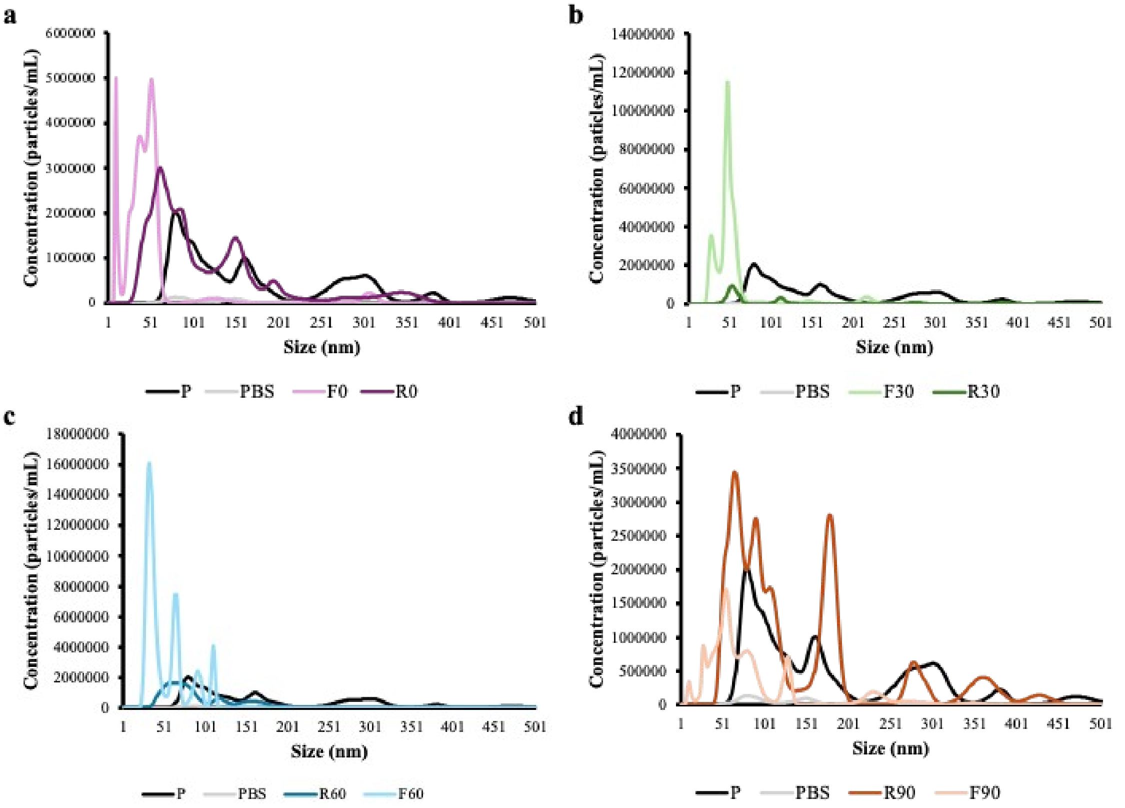

Figure S3: *NTA particle concentration and size distribution analysis of CD9^{+} sEVs in filtrates, retentates, PBS and parents of inlet microchannel orientation to the nanoporous membrane filtration at 0º (a), 30º (b), 60º (c) and 90º (d) in the microfluidic device from n=3 filtration runs.*

S4

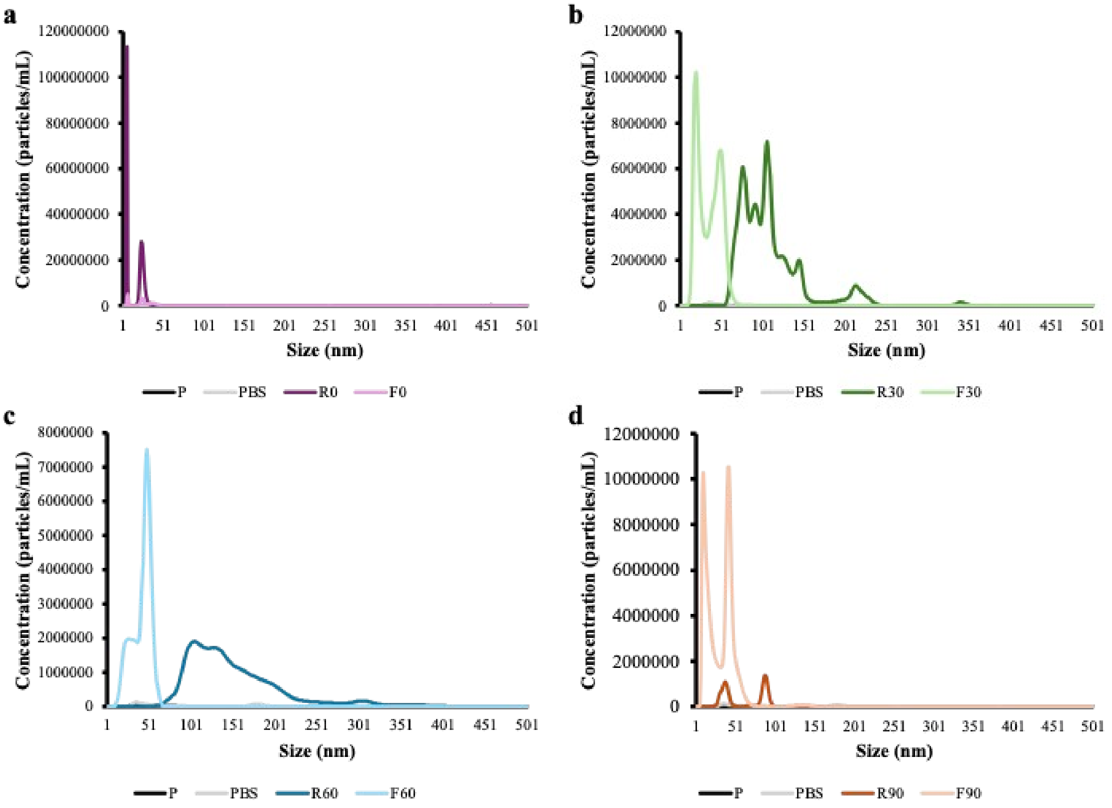

Figure S4: *NTA particle concentration and size distribution analysis of CA125*$^{+}$ *sEVs in filtrates, retentates, PBS and parents of inlet microchannel orientation to the nanoporous membrane filtration at 0º (a), 30º (b), 60º (c) and 90º (d) in the microfluidic device from n=3 filtration runs.*

S5

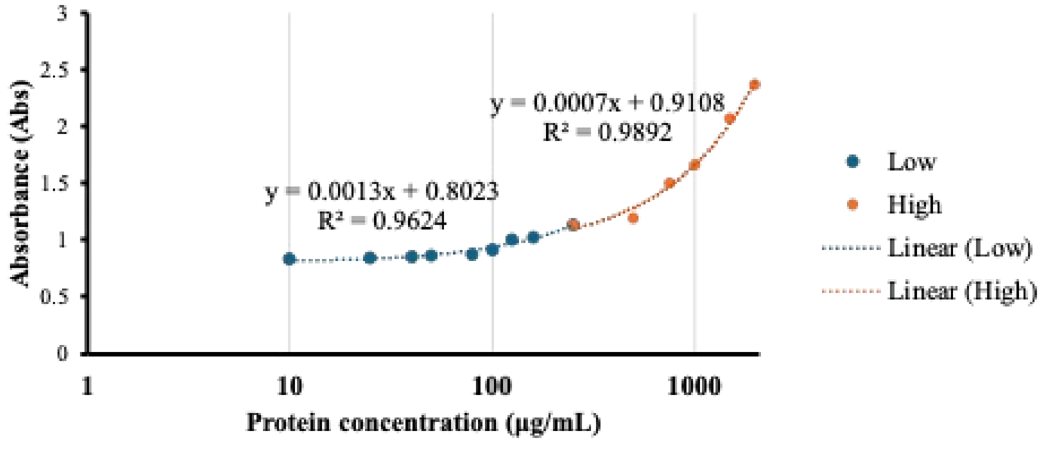

Sample	P	F0	F30	F60	F90	PBS
Abs	**3.40** ± 0.338	**0.19** ± 0.019	**0.17** ± 0.004	**0.22** ± 0.041	**0.17** ± 0.005	**0.14** ± 0.008

Figure S5: *BCA calibration curves using low (micro-BCA) and high albumin concentrations standards and absorbance of samples, P (parent), filtrates (from filtrations at each inlet microchannel orientation to the nanoporous membrane, F0, F30º, F60, F90) and PBS at 562 nm wavelength from at least n=2 readings ± standard deviation.*

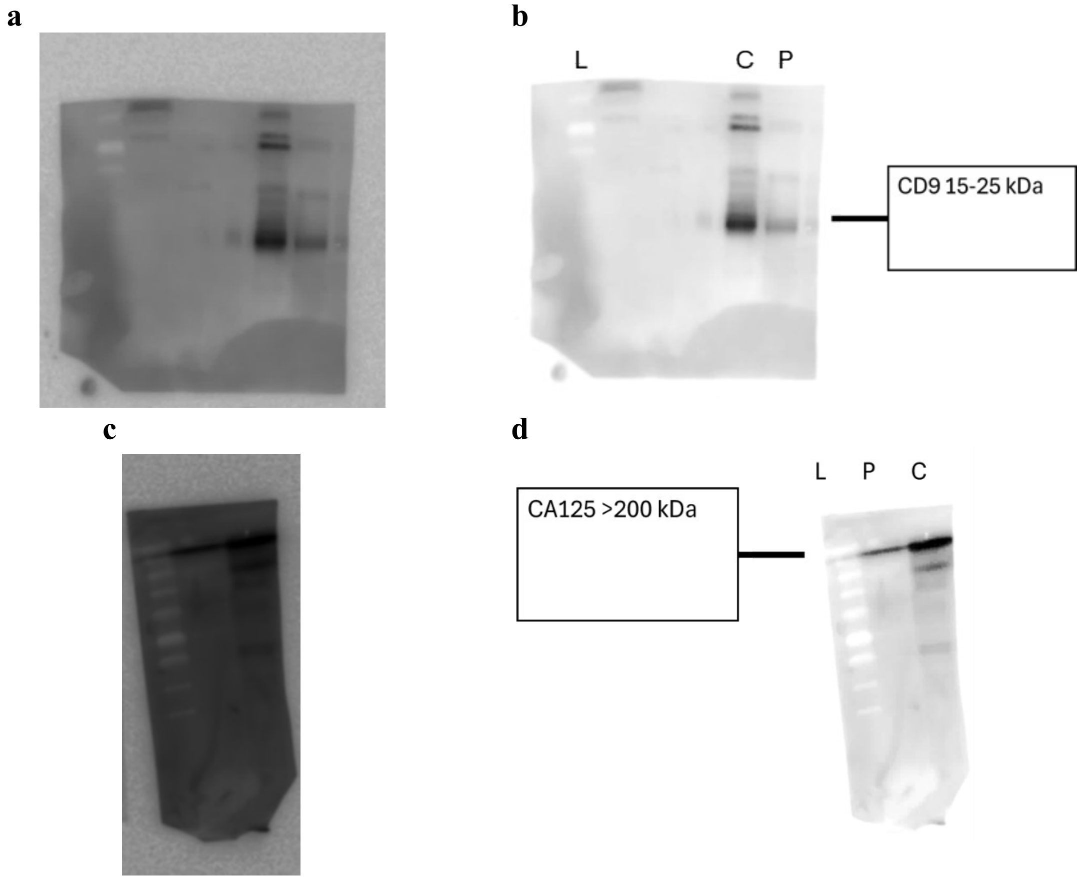

Figure S6: *Western blot images of whole gels of data shown in Figure 4l. Images of gel for CD9 protein (a; b: brightness as in manuscript + labelled) and for CA125 protein (c; d: brightness as in manuscript + labelled). L: Ladder (PageRuler Prestained Protein Ladder ThermoFisher 26616); P: Parent sEV sample; C: OVCAR3 cell lysate.*
